# Supplementary material for: HIV transmission dynamics and population-wide drug resistance in rural South Africa
Source: Nat Commun. 2024 Apr 29;15:3644. doi: 10.1038/s41467-024-47254-z (PMC11059351; doi:10.1038/s41467-024-47254-z)
Supplement: Supplementary file 1 — Supplementary Information [file 41467_2024_47254_MOESM1_ESM.pdf]

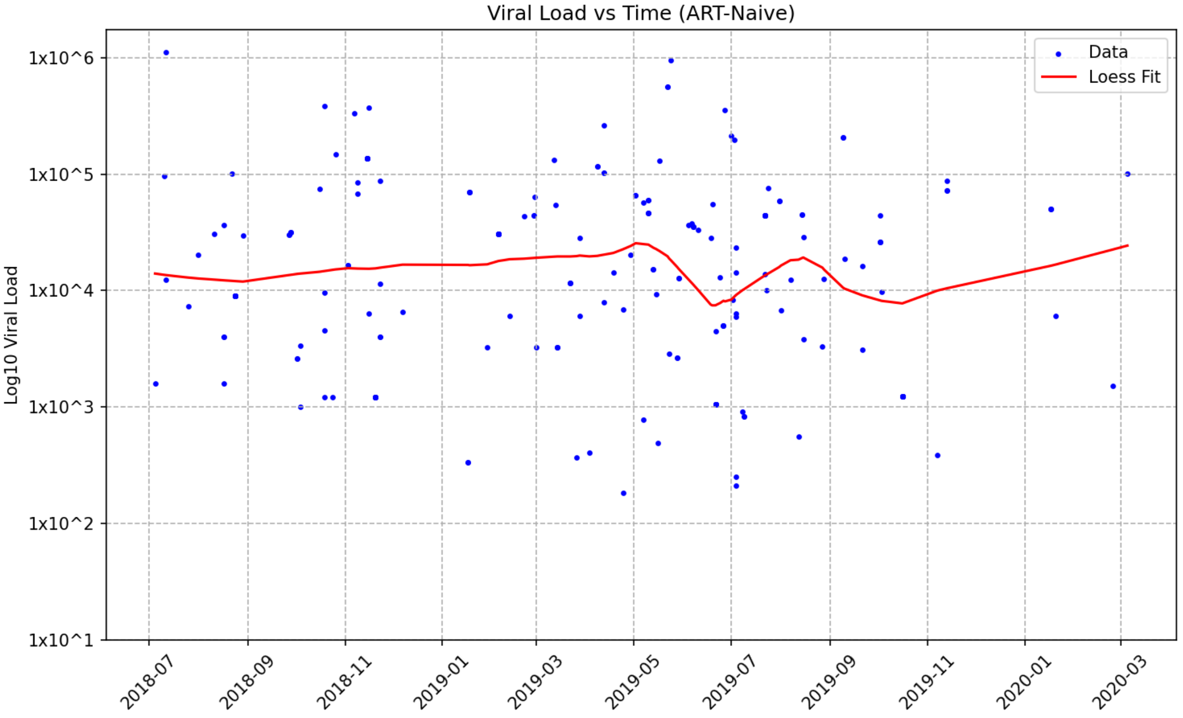

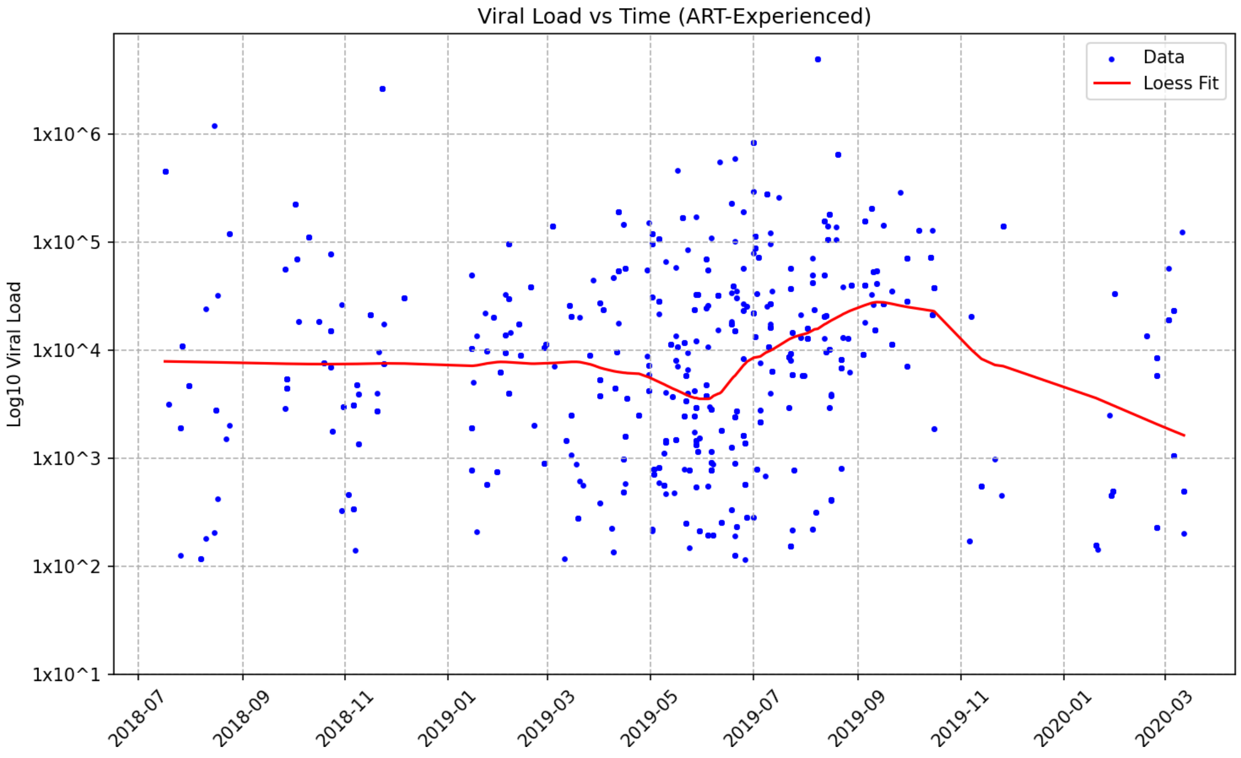

Supplementary Figure 1. Viral abundance relative to sampling. Plots of viral load versus date of sampling for ART-naïve and ART-experienced individuals.

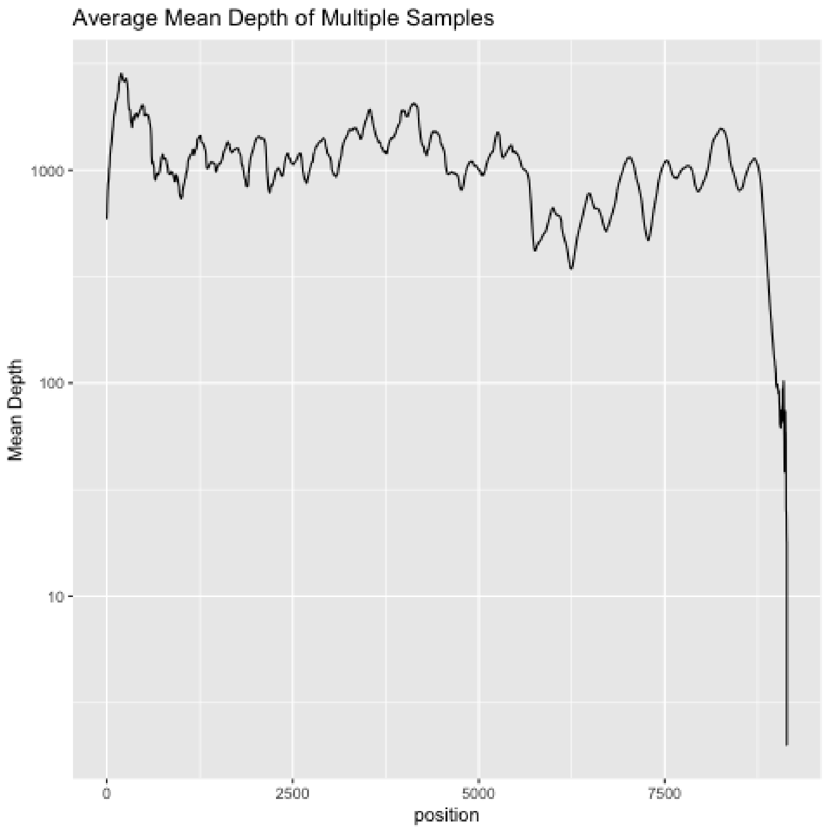

Supplementary Figure 2. Mean coverage of whole-genome sequences used for resistance and phylogenetic linkage analysis. The average mean depth across the whole genome was >500x. Sequencing was performed using Ilumina MiSeq System as detailed in the methods.

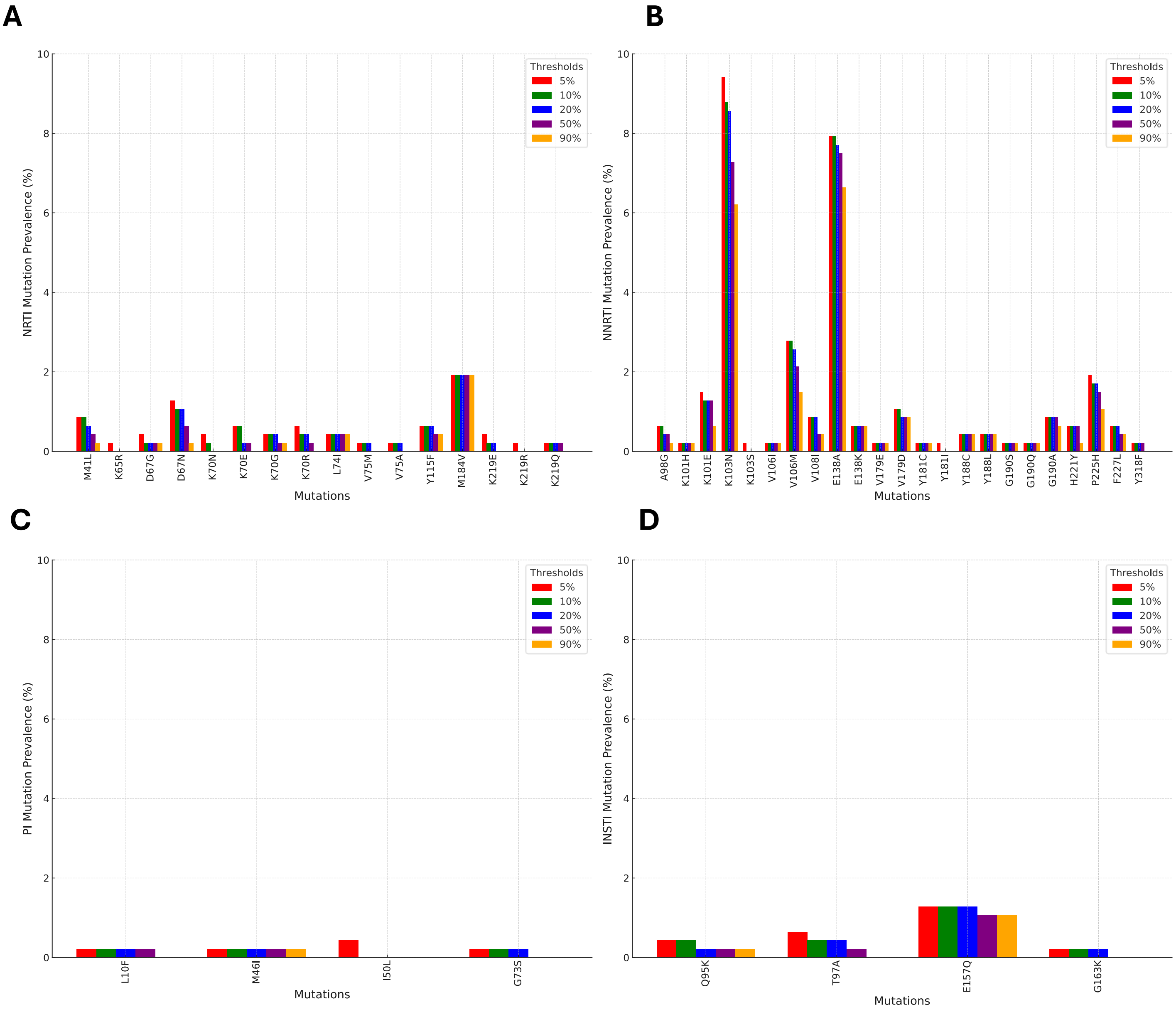

Supplementary Figure 3: Distribution and prevalence of HIV-1 drug resistance-associated mutations amongst 467 ART-naïve individuals. A. Proportion of participants with an NRTI mutation detected at great than the respective thresholds indicated at the top right of panel. **B.** Proportion of participants with a NNRTI mutation detected at greater than the respective thresholds. **C.** Proportion of participants with a PI mutation detected at greater than the respective thresholds. **D.** Proportion of participants with an INSTI mutation. Variant frequency thresholds are 5%, 10%, 20%, 50% and 90%.

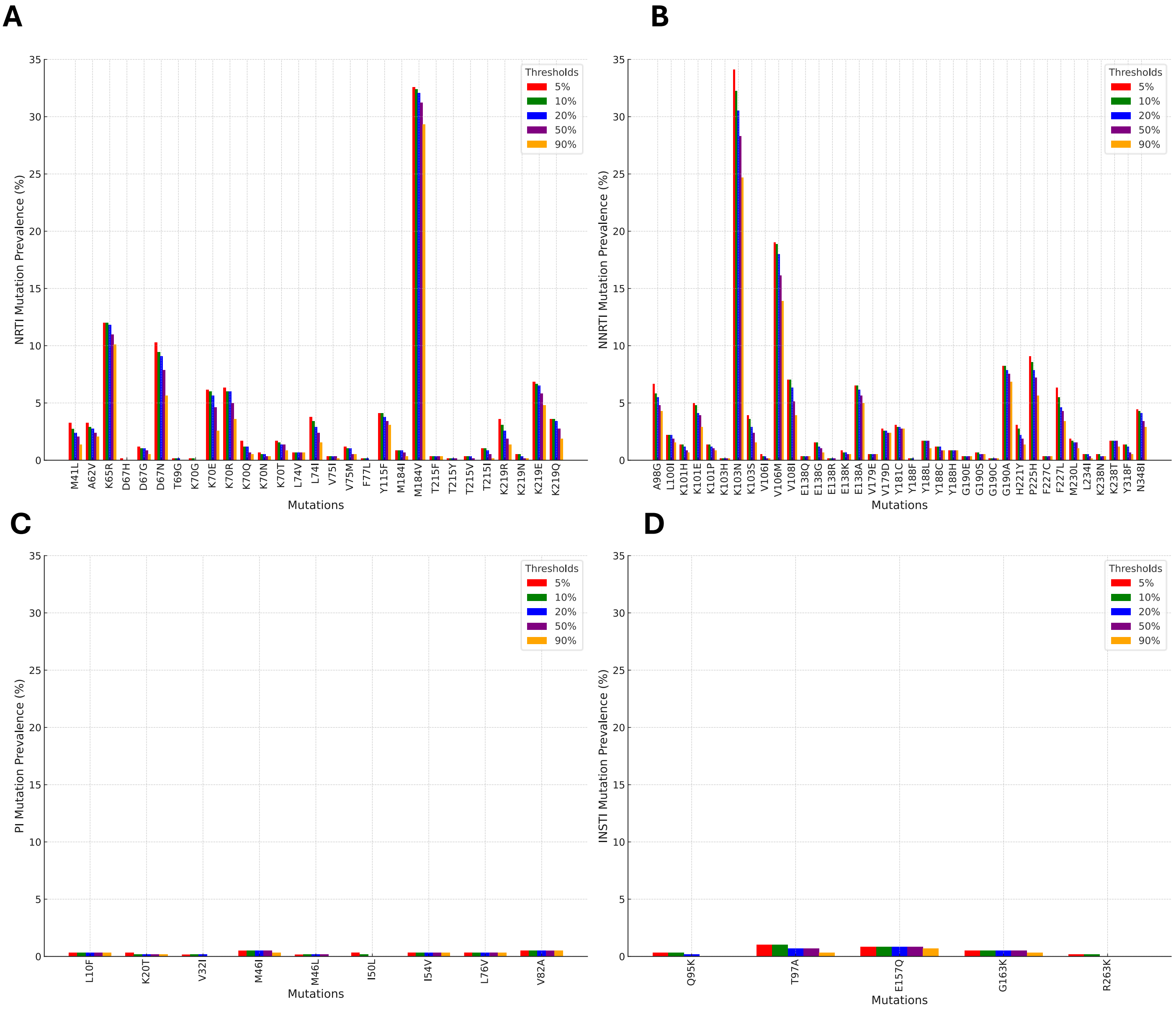

Supplementary Figure 4: Distribution and Prevalence of HIV-1 drug resistance-associated mutations amongst 583 ART-experienced individuals. A. Proportion of participants with an NRTI mutation detected at great than the respective thresholds indicated at the top right of panel. **B.** Proportion of participants with an NNRTI mutation detected at greater than the respective threshold. **C.** Proportion of participants with a PI mutation. **D.** Proportion of participants with an INSTI mutation. Variant frequency thresholds are 5%, 10%, 20%, 50% and 90%.

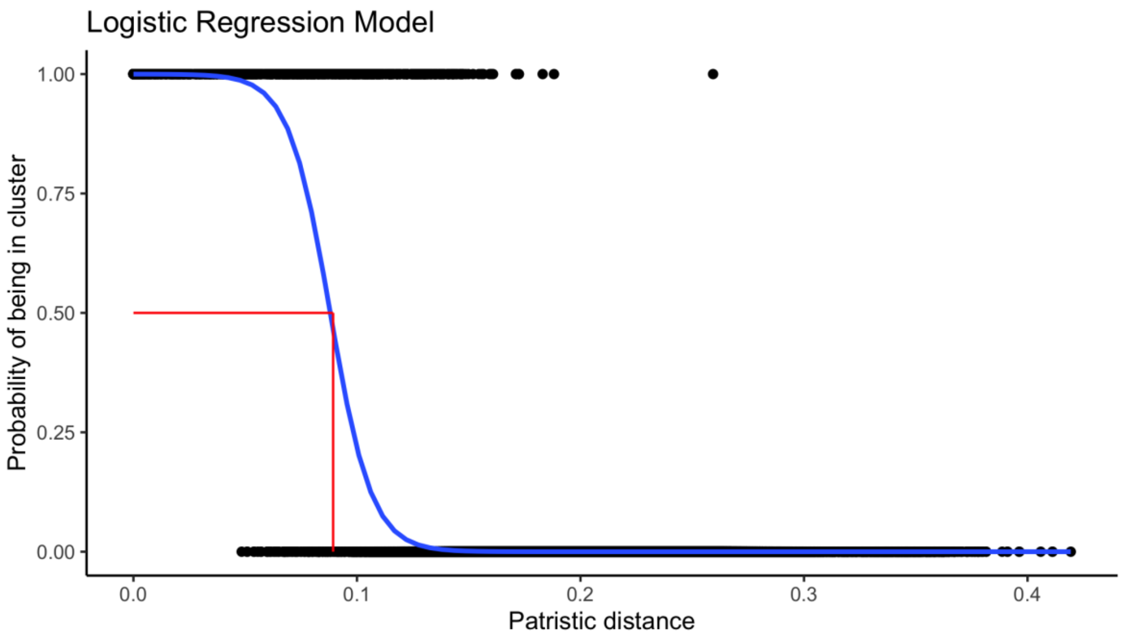

Supplementary Figure 5. Logistic regression model used to refine clusters from ClusterPicker analysis. Data acquired from Cluster Picker was validated through a logit model. The probability of sample presence within a cluster was calculated based on patristic distance between pairs of sequences present on an untimed maximum likelihood phylogenetic tree. R software (v4.1.1) was used to calculate patristic distance using the adephylo function and to perform model assessment using the glm and predict functions. Using the response function with a 50% threshold, values above 0.5 indicating a sequence is within a cluster.
