# Supplementary material for: HIV transmission dynamics and population-wide drug resistance in rural South Africa
Source: Nat Commun. 2024 Apr 29;15:3644. doi: 10.1038/s41467-024-47254-z (PMC11059351; doi:10.1038/s41467-024-47254-z)
Supplement: Supplementary file 3 — Reporting Summary [file 41467_2024_47254_MOESM3_ESM.pdf]

nature portfolio

Corresponding author(s): Ravindra K Gupta

Last updated by author(s): Feb 19, 2024

Reporting Summary

Nature Portfolio wishes to improve the reproducibility of the work that we publish. This form provides structure for consistency and transparency in reporting. For further information on Nature Portfolio policies, see our Editorial Policies and the Editorial Policy Checklist.

Statistics

For all statistical analyses, confirm that the following items are present in the figure legend, table legend, main text, or Methods section.

n/a	Confirmed	
☐	☒	The exact sample size (*n*) for each experimental group/condition, given as a discrete number and unit of measurement
☒	☐	A statement on whether measurements were taken from distinct samples or whether the same sample was measured repeatedly
☒	☐	The statistical test(s) used AND whether they are one- or two-sided<br>*Only common tests should be described solely by name; describe more complex techniques in the Methods section.*
☒	☐	A description of all covariates tested
☒	☐	A description of any assumptions or corrections, such as tests of normality and adjustment for multiple comparisons
☐	☒	A full description of the statistical parameters including central tendency (e.g. means) or other basic estimates (e.g. regression coefficient) AND variation (e.g. standard deviation) or associated estimates of uncertainty (e.g. confidence intervals)
☒	☐	For null hypothesis testing, the test statistic (e.g. *F*, *t*, *r*) with confidence intervals, effect sizes, degrees of freedom and *P* value noted<br>*Give P values as exact values whenever suitable.*
☒	☐	For Bayesian analysis, information on the choice of priors and Markov chain Monte Carlo settings
☒	☐	For hierarchical and complex designs, identification of the appropriate level for tests and full reporting of outcomes
☒	☐	Estimates of effect sizes (e.g. Cohen's *d*, Pearson's *r*), indicating how they were calculated

Our web collection on statistics for biologists contains articles on many of the points above.

Software and code

Policy information about availability of computer code

Data collection	Microsoft Excel 356 v16.79<br>Rstudio v2023.09.1+494<br>Python 3.10<br>ArcGIS Pro 3.1
Data analysis	Custom python and Rscripts were used to produced Figures 2,3, supplementary figure 1 -5 (https://github.com/SteveKemp/Vukuzazi_manuscript)<br>IQTREE v2.2.5 was used to infer phylogenies in figures 4 and supplementary figure 6.<br>Clusterpicker v1.2.5 was used to infer clusters for figures 4 and supplementary figure 6<br>Drawio was used to produce the flowchart in Figure 1<br>Phyloscanner v1.82 was used to infer transmissions between participants (https://github.com/BDI-pathogens/phyloscanner)<br>ArcGIS Pro 3.1 was used to construct the grid and generate the spatial data visualisations included in figure 5.

For manuscripts utilizing custom algorithms or software that are central to the research but not yet described in published literature, software must be made available to editors and reviewers. We strongly encourage code deposition in a community repository (e.g. GitHub). See the Nature Portfolio guidelines for submitting code & software for further information.

Data

Policy information about availability of data

All manuscripts must include a data availability statement. This statement should provide the following information, where applicable:

- Accession codes, unique identifiers, or web links for publicly available datasets
- A description of any restrictions on data availability
- For clinical datasets or third party data, please ensure that the statement adheres to our policy

Due to the potential for stigma and identifiable information involved in potential transmission clusters, accession numbers for these participants are purposefully redacted.

Sequencing data for the entire Vukuzazi/PANGEA cohort are available for download from GenBank, Accession: PRJEB19239 ID: 369369

Research involving human participants, their data, or biological material

Policy information about studies with human participants or human data. See also policy information about sex, gender (identity/presentation), and sexual orientation and race, ethnicity and racism.

Reporting on sex and gender	In table 1, participants are disaggregated into sex, either male or female.
Reporting on race, ethnicity, or other socially relevant groupings	No further disaggregation on race, ethnicity or other socially relevant groupings were made in this study.
Population characteristics	The population was divided into age categories as follows:<br>15-24<br>25-34<br>35-44<br>45-54<br>>55<br><br>The population was also segregated into into ART-naive (n=467) or ART-experienced (n=583). Known ART regimens are also present in the results
Recruitment	All eligible participants from the uMkhanyakude district were invited to participate in this cross-sectional study (n=36,097). Of these, 18041 were enrolled and participated in the study.
Ethics oversight	Ethical clearances were obtained from the University of KwaZulu-Natal Biomedical Research Ethics Committee, the London School of Hygiene & Tropical Medicine Ethics Committee, and the Partners Institutional Review Boards. All participants provided informed consent for HIV testing and ensuing analysis.

Note that full information on the approval of the study protocol must also be provided in the manuscript.

Field-specific reporting

Please select the one below that is the best fit for your research. If you are not sure, read the appropriate sections before making your selection.

☒ Life sciences ☐ Behavioural & social sciences ☐ Ecological, evolutionary & environmental sciences

For a reference copy of the document with all sections, see nature.com/documents/nr-reporting-summary-flat.pdf

Life sciences study design

All studies must disclose on these points even when the disclosure is negative.

Sample size	All eligible participants from the uMkhanyakude district were invited to participate in this cross-sectional study (n=36,097). Of these, 18041 agreed to participate and were enrolled and participated in the study. No sample size calculation was performed, as this was intended to be an all-encompassing health survey of the entire district.<br>Following recruitment, n=6093 participants had a positive HIV ELISA.<br>Sequencing data was available for n=1050 genomes.
Data exclusions	135 samples were excluded due to poor amplification of HIV RNA.<br>47 genomes were excluded due to poor quality control following RNA amplification.
Replication	No replicates were possible due to fixed sequencing data.
Randomization	No randomization took place - particiapnts were in one of two fixed categories: ART-naive or ART-experienced
Blinding	To determine patterns between ART-naive and ART-experienced participants, no blinding took place in this study. Each participant was

allocated a PANGEA identifier and no identifiable data is present in the manuscript.

Reporting for specific materials, systems and methods

We require information from authors about some types of materials, experimental systems and methods used in many studies. Here, indicate whether each material, system or method listed is relevant to your study. If you are not sure if a list item applies to your research, read the appropriate section before selecting a response.

Materials & experimental systems

n/a	Involved in the study
☒	☐ Antibodies
☒	☐ Eukaryotic cell lines
☒	☐ Palaeontology and archaeology
☒	☐ Animals and other organisms
☐	☒ Clinical data
☒	☐ Dual use research of concern
☒	☐ Plants

Methods

n/a	Involved in the study
☒	☐ ChIP-seq
☒	☐ Flow cytometry
☒	☐ MRI-based neuroimaging

Clinical data

Policy information about clinical studies

All manuscripts should comply with the ICMJEguidelines for publication of clinical research and a completedCONSORT checklist must be included with all submissions.

Clinical trial registration	N/A
Study protocol	https://www.thelancet.com/journals/langlo/article/PIIS2214-109X(21)00176-5/fulltext
Data collection	Recruitment was from May 25, 2018 - Nov 28, 2019
Outcomes	N/A

Plants

Seed stocks	*Report on the source of all seed stocks or other plant material used. If applicable, state the seed stock centre and catalogue number. If plant specimens were collected from the field, describe the collection location, date and sampling procedures.*
Novel plant genotypes	*Describe the methods by which all novel plant genotypes were produced. This includes those generated by transgenic approaches, gene editing, chemical/radiation-based mutagenesis and hybridization. For transgenic lines, describe the transformation method, the number of independent lines analyzed and the generation upon which experiments were performed. For gene-edited lines, describe the editor used, the endogenous sequence targeted for editing, the targeting guide RNA sequence (if applicable) and how the editor was applied.*
Authentication	*Describe any authentication procedures for each seed stock used or novel genotype generated. Describe any experiments used to assess the effect of a mutation and, where applicable, how potential secondary effects (e.g. second site T-DNA insertions, mosiacism, off-target gene editing) were examined.*
