# Supplementary material for: HIV transmission dynamics and population-wide drug resistance in rural South Africa
Source: Nat Commun. 2024 Apr 29;15:3644. doi: 10.1038/s41467-024-47254-z (PMC11059351; doi:10.1038/s41467-024-47254-z)
Supplement: Supplementary file 4 — 41467_2024_47254_MOESM4_ESM [file 41467_2024_47254_MOESM4_ESM.docx]

**Vukuzazi Study Team**

***Contributing authors**

| **Name** | **Affiliation** | **Role** |
| --- | --- | --- |
| *Deenan Pillay | See byline at beginning of paper | Principal Investigator (2017-2019) |
| *Willem Hanekom | See byline at beginning of paper | Principal Investigator (2019-present) |
| *Emily Wong | See byline at beginning of paper | Co-Principal Investigator |
| *Mark Siedner | See byline at beginning of paper | Co-Principal Investigator |
| Olivier Koole | African Health Research Institute | Co-Principal Investigator (2017-2019) |
| *Thumbi Ndung’u | See byline at beginning of paper | Co-investigator |
| Thandeka Khoza | African Health Research Institute | Co-investigator (2019-present) |
| Kobus Herbst | African Health Research Institute | Co-investigator |
| Kathy Baisley | African Health Research Institute | Co-investigator |
| Janet Seeley | African Health Research Institute | Co-investigator |
| Alison Grant | African Health Research Institute | Co-investigator |
| Resign Gunda | African Health Research Institute | Programme Manager |
| Ashmika Surujdeen | African Health Research Institute | Study Coordinator |
| Theresa Smit | African Health Research Institute | Head: Diagnostic Research |
| Dickman Gareta | African Health Research Institute | Head: Research Data Management |
| Day Munatsi | African Health Research Institute | Head: Research Data Systems |
| Ngcebo Mhlongo | African Health Research Institute | Study Physician |
| Tshwaraganang Modise | African Health Research Institute | Research Data Manager |
| Jaco Dreyer | African Health Research Institute | Senior Research Data Manager |
| Siyabonga Nxumalo | African Health Research Institute | Research Data Manager |
| Stephen Olivier | African Health Research Institute | Statistician |
| Gregory Ording- Jespersen | African Health Research Institute | Laboratory Data Supervisor |
| Innocentia Mpofana | Africa Health Research Institute | Diagnostic Laboratory Manager |
| Khadija Khan | Africa Health Research Institute | Biorepository Manager |
| Zizile Sikhosana | Africa Health Research Institute | Somkhele Laboratory Supervisor |
| Sashen Moodley | Africa Health Research Institute | Microbiology Laboratory Supervisor |
| Hollis Shen | Africa Health Research Institute | Head: Exploratory Research Division |
| Philippa Mathews | Africa Health Research Institute | Clinical Governance |
| Nompilo Buthelezi | Africa Health Research Institute | Training Coordinator |
| Hlolisile Khumalo | Africa Health Research Institute | Nursing Manager |
| Sanah Bucibo | Africa Health Research Institute | Professional Nurse |
| Nozipho Mbonambi | Africa Health Research Institute | Professional Nurse |
| Hloniphile Ngubane | Africa Health Research Institute | Professional Nurse |
| Thokozani Simelane | Africa Health Research Institute | Professional Nurse |
| Khanyisani Buthelezi | Africa Health Research Institute | Professional Nurse |
| Sphiwe Ntuli | Africa Health Research Institute | Professional Nurse |
| Nombuyiselo Zondi | Africa Health Research Institute | Professional Nurse |
| Siboniso Nene | Africa Health Research Institute | Professional Nurse |
| Bongumenzi Ndlovu | Africa Health Research Institute | Enrolled Nurse |
| Talente Ntimbane | Africa Health Research Institute | Enrolled Nurse |
| Mbali Mbuyisa | Africa Health Research Institute | Enrolled Nurse |
| Xolani Mkhize | Africa Health Research Institute | Enrolled Nurse |
| Melusi Sibiya | Africa Health Research Institute | Enrolled Nurse |
| Ntombiyenkosi Ntombela | Africa Health Research Institute | Enrolled Nurse |
| Mandisi Dlamini | Africa Health Research Institute | Enrolled Nurse |
| Hlobisile Chonco | Africa Health Research Institute | Enrolled Nurse |
| Hlengiwe Dlamini | Africa Health Research Institute | Enrolled Nurse |
| Doctar Mlambo | Africa Health Research Institute | Enrolled Nurse |
| Nonhlanhla Mzimela | Africa Health Research Institute | Enrolled Nurse |
| Zinhle Buthelezi | Africa Health Research Institute | Enrolled Nurse |
| Zinhle Mthembu | Africa Health Research Institute | Enrolled Nurse |
| Thokozani Bhengu | Africa Health Research Institute | Enrolled Nurse |
| Sandile Mthembu | Africa Health Research Institute | Enrolled Nurse |
| Phumelele Mthethwa | Africa Health Research Institute | Enrolled Nurse |
| Zamashandu Mbatha | Africa Health Research Institute | Enrolled Nurse |
| Welcome Petros Mthembu | Africa Health Research Institute | Enrolled Nurse |
| Anele Mkhwanazi | Africa Health Research Institute | Clinical Research Assistant Supervisor |
| Mandlakayise Zikhali | Africa Health Research Institute | Clinical Research Assistant Supervisor |
| Phakamani Mkhwanazi | Africa Health Research Institute | Clinical Research Assistant |
| Ntombiyenhlanhla Mkhwanazi | Africa Health Research Institute | Clinical Research Assistant |
| Rose Myeni | Africa Health Research Institute | Clinical Research Assistant |
| Fezeka Mfeka | Africa Health Research Institute | Clinical Research Assistant |
| Hlobisile Gumede | Africa Health Research Institute | Clinical Research Assistant |
| Nonceba Mfeka | Africa Health Research Institute | Clinical Research Assistant |
| Ayanda Zungu | Africa Health Research Institute | Clinical Research Assistant |
| Nonhlanhla Mfekayi | Africa Health Research Institute | Clinical Research Assistant |
| Smangaliso Zulu | Africa Health Research Institute | Clinical Research Assistant |
| Mzamo Buthelezi | Africa Health Research Institute | Clinical Research Assistant |
| Senzeni Mkhwanazi | Africa Health Research Institute | Clinical Research Assistant |
| Mlungisi Dube | Africa Health Research Institute | Clinical Research Assistant |
| Hosea Kambonde | iMarketing Consultants | IT Systems Developer |
| Lindani Mthembu | Africa Health Research Institute | Information Technology Assistant |
| Seneme Mchunu | Africa Health Research Institute | Information Technology Assistant |
| Sibahle Gumbi | Africa Health Research Institute | Research Admin Assistant |
| Tumi Madolo | Africa Health Research Institute | Research Data Manager |
| Thengokwakhe Nkosi | Africa Health Research Institute | Driver |
| Sibusiso Mkhwanazi | Africa Health Research Institute | Driver |
| Sibusiso Nsibande | Africa Health Research Institute | Driver |
| Mpumelelo Steto | Africa Health Research Institute | Driver |
| Sibusiso Mhlongo | Africa Health Research Institute | Driver |
| Velile Vellem | Aurum Innova (Pty) Ltd | Driver |
| Pfarelo Tshivase | Aurum Innova (Pty) Ltd | Driver |
| Jabu Kwinda | Aurum Innova (Pty) Ltd | Driver |
| Bongani Magwaza | Africa Health Research Institute | General Worker |
| Siyabonga Nsibande | Africa Health Research Institute | General Worker |
| Skhumbuzo Mthombeni | Africa Health Research Institute | General Worker |
| Sphiwe Clement Mthembu | Africa Health Research Institute | General Worker |
| Antony Rapulana | Africa Health Research Institute | Laboratory Technologist |
| Jade Cousins | Africa Health Research Institute | Laboratory Technologist |
| Thabile Zondi | Africa Health Research Institute | Laboratory Technologist |
| Nagavelli Padayachi | Africa Health Research Institute | Laboratory Technologist |
| Freddy Mabetlela | Africa Health Research Institute | Laboratory Technologist |
| Simphiwe Ntshangase | Africa Health Research Institute | Laboratory Technician/LIMS Administrator |
| Nomfundo Luthuli | Africa Health Research Institute | Laboratory Technician |
| Sithembile Ngcobo | Africa Health Research Institute | Laboratory Technologist |
| Kayleen Brien | Africa Health Research Institute | Laboratory Technologist |
| Sizwe Ndlela | Africa Health Research Institute | Laboratory Technician |
| Nomfundo Ngema | Africa Health Research Institute | Laboratory Technician |
| Nokukhanya Ntshakala | Africa Health Research Institute | Laboratory Technician |
| Anupa Singh | Africa Health Research Institute | Laboratory Technician |
| Rochelle Singh | Africa Health Research Institute | Laboratory Technician |
| Logan Pillay | Africa Health Research Institute | Laboratory Technician |
| Kandaseelan Chetty | Africa Health Research Institute | Laboratory Technician |
| Ashentha Govender | Africa Health Research Institute | Laboratory Technician |
| Pamela Ramkalawon | Africa Health Research Institute | Laboratory Research Technician |
| Nondumiso Mabaso | Africa Health Research Institute | Laboratory Intern |
| Kimeshree Perumal | Africa Health Research Institute | Laboratory Intern |
| Senamile Makhari | Africa Health Research Institute | Biorepository Laboratory Technician |
| Nondumiso Khuluse | Africa Health Research Institute | Biorepository Laboratory Technician |
| Nondumiso Zitha | Africa Health Research Institute | Biorepository Research Assistant |
| Hlengiwe Khathi | Africa Health Research Institute | Biorepository Research Assistant |
| Mbuti Mofokeng | Africa Health Research Institute | Clinical Specimen Driver/Laboratory Assistant |
| Nomathamsanqa Majozi | Africa Health Research Institute | Public Engagement |
| Nceba Gqaleni | Africa Health Research Institute | Public Engagement |
| Hannah Keal | Africa Health Research Institute | Communications |
| Phumla Ngcobo | Africa Health Research Institute | Communications |
| Costa Criticos | Africa Health Research Institute | Operational Oversight |
| Raynold Zondo | Africa Health Research Institute | Operational Oversight |
| Dilip Kalyan | Africa Health Research Institute | Operational Oversight |
| Clive Mavimbela | Africa Health Research Institute | Operational Oversight |
| Anand Ramnanan | Africa Health Research Institute | Procurement |
| Sashin Harilall | Africa Health Research Institute | Grants Office |
| Kennedy Nyamande | University of KwaZulu-Natal | Pulmonology Consultant |
| Jaikrishna Kalideen | Perumal and Partners Radiologist Inc | Radiologist |
| Ramesh Jackpersad | Jacpersad and Partners Inc | Radiologist |
| Kgaugelo Moropane | Aurum Innova (Pty) Ltd | Radiographer |
| Boitsholo Mfolo | Aurum Innova (Pty) Ltd | Radiographer |
| Khabonina Malomane | Aurum Innova (Pty) Ltd | Radiographer |
